# The CcmC–CcmE interaction during cytochrome *c* maturation by System I is driven by protein–protein and not protein–heme contacts

**DOI:** 10.1074/jbc.RA118.005024

**Published:** 2018-09-11

**Authors:** Shevket H. Shevket, Diego Gonzalez, Jared L. Cartwright, Colin Kleanthous, Stuart J. Ferguson, Christina Redfield, Despoina A. I. Mavridou

**Affiliations:** From the ‖ MRC Centre for Molecular Bacteriology and Infection, Department of Life Sciences, Imperial College London, Kensington, London SW7 2DD, United Kingdom,; the ‡ Department of Biochemistry, University of Oxford, South Parks Road, Oxford OX1 3QU, United Kingdom,; the § Department of Fundamental Microbiology, Faculty of Biology and Medicine, University of Lausanne, Quartier UNIL/Sorge, Lausanne, Switzerland,; the ¶ Bioscience Technology Facility, Department of Biology, University of York, Wentworth Way, York YO10 5DD, United Kingdom

**Keywords:** cytochrome c, heme, post-translational modification (PTM), Gram-negative bacteria, nuclear magnetic resonance (NMR), CcmC, CcmE, cytochrome c maturation, protein-protein interactions, System I

## Abstract

Cytochromes *c* are ubiquitous proteins, essential for life in most organisms. Their distinctive characteristic is the covalent attachment of heme to their polypeptide chain. This post-translational modification is performed by a dedicated protein system, which in many Gram-negative bacteria and plant mitochondria is a nine-protein apparatus (CcmA–I) called System I. Despite decades of study, mechanistic understanding of the protein–protein interactions in this highly complex maturation machinery is still lacking. Here, we focused on the interaction of CcmC, the protein that sources the heme cofactor, with CcmE, the pivotal component of System I responsible for the transfer of the heme to the apocytochrome. Using *in silico* analyses, we identified a putative interaction site between these two proteins (residues Asp^47^, Gln^50^, and Arg^55^ on CcmC; Arg^73^, Asp^101^, and Glu^105^ on CcmE), and we validated our findings by *in vivo* experiments in *Escherichia coli*. Moreover, employing NMR spectroscopy, we examined whether a heme-binding site on CcmE contributes to this interaction and found that CcmC and CcmE associate via protein–protein rather than protein–heme contacts. The combination of *in vivo* site-directed mutagenesis studies and high-resolution structural techniques enabled us to determine at the residue level the mechanism for the formation of one of the key protein complexes for cytochrome *c* maturation by System I.

## Introduction

*c-*Type cytochromes have a central role in all kingdoms of life due to their involvement in essential cellular processes (1, 2). Primarily, they function as electron carriers in both aerobic and anaerobic respiration. In eukaryotes, mitochondrial cytochrome *c* shuttles electrons from the *bc*_1_ complex to cytochrome *c* oxidase during aerobic respiration, ultimately generating a proton electrochemical gradient across the membrane, which drives ATP synthesis (3). In bacteria, where respiration can be performed by several different pathways, cytochromes *c* usually have a role in quinol-oxidizing steps (4). In addition to their classical electron transport function in respiration and photosynthesis, *c*-type cytochromes have also been assigned roles in other important biological processes, like triggering apoptosis in mammalian cells (5), assembling the mitochondrial cytochrome *c* oxidase (6), and scavenging H_2_O_2_ in yeast and Gram-negative bacteria (7). They are found in most organisms, and, in prokaryotes especially, they exhibit an impressive range of shapes, sizes, and heme content (8). Unlike other commonly encountered heme-containing proteins, the distinguishing characteristic of cytochromes *c* is that at least one of their heme groups is always covalently and stereospecifically bound to two cysteines of a conserved C*XX*CH motif (9). This attachment occurs as a post-translational modification, after the apocytochrome (protein without heme) is translocated to the biological compartment in which it will function as a holoenzyme (protein with heme).

**Figure 1.**
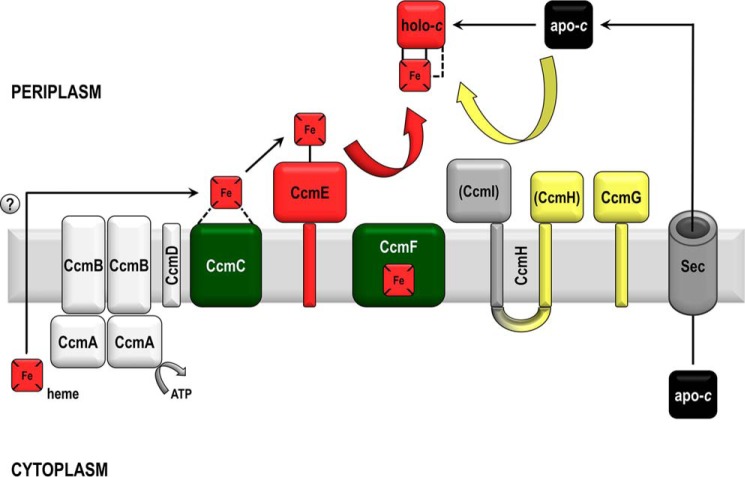
**Cytochrome *c* biogenesis System I is a multiprotein apparatus (CcmA-I) predominantly located in the inner membrane and periplasm of Gram-negative bacteria.** Shown is a *schematic representation* of its main components based on the model organism *E. coli*. Proteins that are known to bind heme (*red squares*) covalently or noncovalently are depicted in *red* or *green*, respectively, whereas proteins that are thought to be involved in reductant provision are depicted in *yellow*. Apocytochrome *c* (in *black*) is synthesized in the cytoplasm and transported across the cytoplasmic membrane by the Sec apparatus (65). Heme is also synthesized in the cytoplasm, but its transport route to the point of assembly is unknown (indicated by a *question mark*). In most System I–containing organisms, CcmH and CcmI are two different membrane-anchored proteins; in *E. coli*, CcmH and CcmI are fused into one protein, usually referred to as CcmH.

Despite the relatively simple chemical nature of the covalent bonds in cytochromes *c*, the maturation process itself is very elaborate, as it relies on multiple complex steps, including protein translocation and folding, post-translational modification, redox homeostasis, and cofactor acquisition, transfer, and insertion into the polypeptide chain. Unsurprisingly, living organisms employ several different cytochrome *c* maturation (Ccm)^4^ systems to perform this task (10–14). System I, found in most Gram-negative bacteria and plant and protozoal mitochondria, is undoubtedly the most complex of the Ccm apparatuses, possibly accounting for the vast diversity of bacterial cytochromes *c* and the fact that often one bacterial species will encode several entirely different *c*-type cytochromes (8). System I comprises nine proteins, CcmA–I, which in Gram-negative bacteria are located in the inner membrane or have protein domains oriented into the periplasm; CcmA is an exception, and it is found on the cytoplasmic side of the inner membrane. A schematic representation of System I is shown in Fig. 1.

Essential roles for almost all proteins of System I have been assigned (15–18), and their function has been extensively reviewed (12, 19–21). More recently, a modular approach has been adopted whenever System I is described (12, 14, 21). The first module, CcmA–E, deals with heme transport and provision. Although the transport route of the heme to the periplasm for cytochrome *c* biogenesis purposes remains unknown, CcmC is thought to be the protein responsible for sourcing the heme for System I (22, 23). Along with CcmD, heme-bound CcmC forms a complex with the heme chaperone CcmE (22–24). In the environment of this protein complex, CcmE binds the heme covalently through a conserved histidine residue (25). The CcmC–heme–CcmE complex is subsequently resolved through the ATPase activity of CcmA and presumably conformational rearrangements involving CcmB (26, 27). CcmG and CcmH (which in *Escherichia coli*, the model organism for studying System I, is fused with CcmI; Fig. 1) form a second module responsible for thio-redox control of the substrate apocytochrome. This is essential because the action of the oxidase DsbA on the two cysteines of the C*XX*CH apocytochrome motif needs to be counteracted before heme attachment (28–30). Finally, CcmF, CcmH, and CcmI form the third System I module. These proteins are involved in binding the apocytochrome and bringing it into contact with holo-CcmE in order for heme to be transferred and stereospecific heme attachment to take place (31–34). CcmI is proposed to have a chaperone role binding the apocytochrome (34), and CcmF, which contains its own noncovalently bound heme (22), is thought to be the “heme lyase” of System I, although a mechanism has not yet been proposed.

Protein–protein interactions between the components of System I must be key to its cytochrome *c* biogenesis function. Currently, it is not clear whether the proteins of this system form one large supercomplex or are organized in several smaller modules as described above. Both hypotheses are plausible and have been proposed in the literature (21, 35). Several interactions between proteins of System I have been experimentally validated (recently reviewed comprehensively by Verissimo and Daldal (21)), as have both covalent and noncovalent protein–heme interactions (22, 24, 36). However, the lack of structural information on this system is striking (only the structures of apo-CcmE (37), CcmG (38), CcmH (39), and an analog of CcmI (40) have been determined). Therefore, mechanistic details on protein–protein interactions, even pairwise associations between known protein partners, remain largely uncharacterized.

In this study, we focus on the interaction of CcmC with CcmE, which leads to holo-CcmE formation. This protein pair is one of the most studied in bacterial cytochrome *c* biogenesis. CcmC and CcmE, along with the small polypeptide CcmD (Fig. 1), are known to associate in a stable complex, which, when purified, contains heme (22, 24). Although CcmC does not bind heme stably in the absence of CcmE, the formation and isolation of the CcmC–heme–CcmE complex is not dependent on the heme-binding histidine of CcmE (22). The exact role of CcmD in this process remains enigmatic, but it has been shown that whereas it is not required for covalent attachment of heme to CcmE, its presence is essential for release of the holo-product (41). Despite several studies on these protein partners, there are still many unknowns regarding the association of CcmC with CcmE. What drives this interaction? Do CcmC and CcmE bind to each other through specific residues? Or is the formation of the CcmC–heme–CcmE complex mediated by the heme that is bound on CcmC and a heme-binding site on CcmE? Here, we employ *in silico* analyses to look for an interaction site between the two proteins and validate our findings in *E. coli* using site-directed mutagenesis in an *in vivo* experimental system. We complement our *in vivo* experiments with NMR spectroscopy to study the interaction of apo-CcmE with heme *in vitro* and to assess the presence of a heme-binding site on this protein. This combined approach allows us to answer all of the above questions in a definitive manner and to elucidate the mechanism of one of the crucial steps of cytochrome *c* maturation by System I.

## Results

### Covariance and bioinformatic analyses identify a putative CcmC–CcmE interaction site

In 2014, Ovchinnikov *et al.* (42) found that amino acid coevolution analysis could be used to predict whether two proteins interact. More specifically, it was shown that residue–residue covariance occurs frequently between physically interacting protein pairs and rarely between noninteracting proteins. Within this study, a prediction of interacting protein partners of unknown structure was performed for the *E. coli* proteome, and a particular pair of residues, Gln^49^ in CcmC and Arg^104^ in CcmE, scored the highest in this analysis (see Fig. 3 of Ovchinnikov et al. (42)); neither CcmC nor CcmE was highlighted in any of the other predicted interactions. In the context of the Ovchinnikov *et al.* study (42), this result was relatively unsurprising because CcmC and CcmE are known to interact (22, 24). However, as the mechanism of interaction between these two proteins remains unknown, this prediction was exciting because it indicated a high probability for the presence of a CcmC–CcmE interaction site involving these two residues.

We sought to assess the role of CcmC Gln^49^ and CcmE Arg^104^ by using site-directed mutagenesis on the entire *E. coli* System I operon and examining the levels of holo-CcmE and holocytochrome *c* matured *in vivo*. We initially generated single-amino acid substitutions, where each of the two amino acids was replaced by an alanine. Surprisingly these mutations did not seem to have a very strong effect on System I function. Whereas holo-CcmE levels were slightly elevated for the Q49A-CcmC variant, they remained unaffected for the R104A-CcmE variant. In addition, holocytochrome *c* (holo-*c*_550_) levels were comparable with the WT System I for both variants (*lanes 1–3* in Fig. 3*A*; a full description of this figure will follow). Taken together, these results suggest that these residues are not essential for the formation of a functional CcmC–CcmE complex.

Reasoning that the amino acids involved in the interaction surface of the two proteins could be near the covarying pair, we turned to bioinformatics. We searched in bacterial and archaeal representative proteomes for homologs of *E. coli* CcmC and CcmE and identified conserved amino acids that could mediate protein–protein interactions and were in the same part of the proteins as the residues identified by the covariance analysis. For *E. coli* CcmC, Asp^47^ (in ∼87% of cases, it is either an Asp or a Glu), Gln^50^ (∼65% conservation), and Arg^55^ (it is either an Arg or a Lys) were plausible candidates (Fig. S1). For *E. coli* CcmE, Asp^101^ (in ∼64% of cases, it is either an Asp or a Glu) and Glu^105^ (in ∼84% of cases, it is either a Glu or an Asp) were chosen (Fig. S2). The rationale behind considering these amino acids was their high conservation and their ability to form electrostatic or hydrogen bond interactions. As only two putative interacting amino acids could be selected for CcmE, we also examined the solution NMR structure of this protein (37) and searched for a third candidate, ideally one that would be positively charged or capable of forming a hydrogen bond with a negatively charged residue. We based this on the nature of the amino acids we had already identified on CcmC and we hypothesized that, although we could not assign specific amino acid pairs, most likely Gln^50^ and Arg^55^ of CcmC would be interacting with Asp^101^ and Glu^105^ of CcmE. Thus, we were looking for a partner for Asp^47^. We identified CcmE Arg^73^ (in ∼58% of cases, it is either an Arg or a Lys) as a potential candidate. Although this residue is not near Arg^104^ in the primary sequence (Fig. S2), it is located near it and near Asp^101^ and Glu^105^ in the 3D structure of apo-CcmE (see Fig. 4*B*).

### Three pairs of amino acids mediate the CcmC–CcmE interaction and are collectively essential for holo-CcmE and holocytochrome c formation

**Figure 2.**
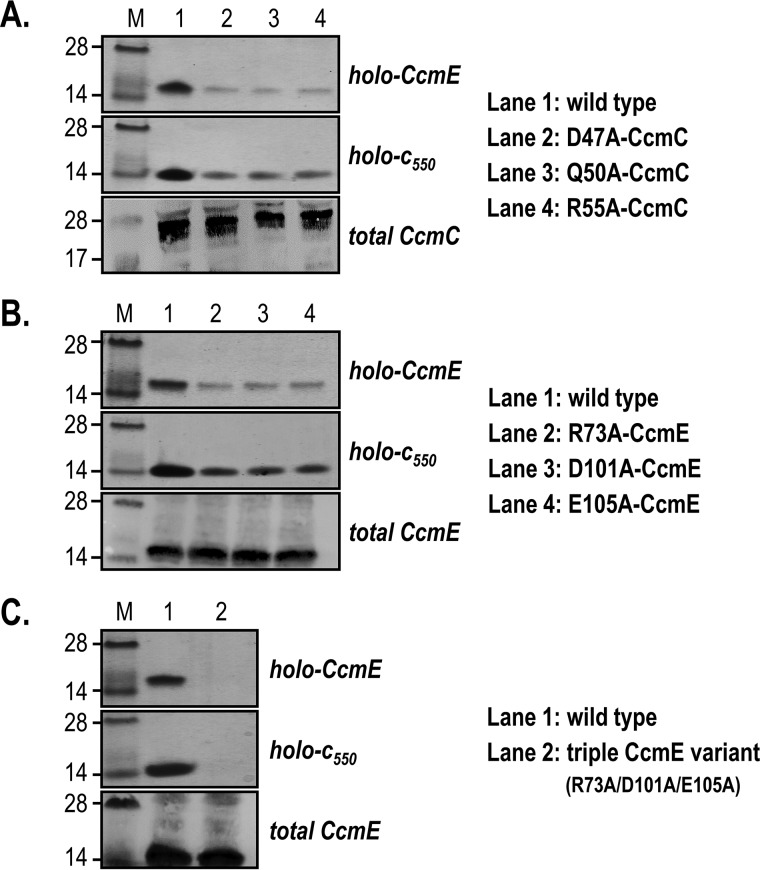
**The putative interaction site between CcmC and CcmE can be validated by *in vivo* experiments in *E. coli*.** *A*, replacement of Asp^47^, Gln^50^, or Arg^55^ by alanine in CcmC leads to decrease in holo-CcmE and holo-*c*_550_. *B*, replacement of Arg^73^, Asp^101^, or Glu^105^ by alanine in CcmE leads to a decrease in holo-CcmE and holo-*c*_550_. *C*, replacement of all putative CcmE interaction residues (Arg^73^, Asp^101^, and Glu^105^) by alanines abrogates holo-CcmE and holo-*c*_550_ formation. Levels of total CcmC and CcmE are not affected by any of the mutations. For all *panels*, representative SDS-PAGE of cell membranes or periplasmic fractions stained for proteins containing covalently bound heme (*top* and *middle gels* of each *panel*) and immunoblotted to detect total CcmC or CcmE amounts (*bottom gel* of each *panel*) are shown. Molecular weight markers (*M*) are on the *left*.

The presence of an interaction site between *E. coli* CcmC and CcmE was tested *in vivo* using variant System I operons where each of the six identified residues (Asp^47^, Gln^50^, and Arg^55^ on CcmC; Arg^73^, Asp^101^, and Glu^105^ on CcmE) was replaced by an alanine. We observed identical results for the maturation of holo-CcmE and holo-*c*_550_ for all tested variant operons. Holo-CcmE decreased significantly (*lanes 2–4* in Fig. 2 (*A* and *B*), *top gel strip*) compared with the level matured by the WT System I (seen in *lane 1*). Holo-*c*_550_ amounts were also significantly reduced (*lanes 2–4* in Fig. 2 (*A* and *B*), *middle gel strip*), reflecting the decrease in holo-CcmE. Total CcmC (Fig. 2*A*, *bottom gel strip*) and CcmE (Fig. 2*B*, *bottom gel strip*) levels in all variants were assessed by immunoblotting and were found to be unaffected by the amino acid replacements.

The results from the single-amino acid substitutions indicate involvement of these residues in the interaction of CcmC with CcmE. To assess their collective role, we investigated whether removal of all three of them on one of the protein partners would abolish the residual holo-CcmE amount obtained with the single variants (Fig. 2 (*A* and *B*), *top*). We carried this out by mutating residues in CcmE (R73A/D101A/E105A mutation; Fig. 2*C*) and found that production of holo-CcmE and holo-*c*_550_ was entirely abolished (*lane 2* in Fig. 2*C*, *top* and *middle gel strips*), whereas the total CcmE amount remained unaffected (*lane 2* in Fig. 2*C*, *bottom gel strip*). Thus, we can conclude that the interactions between Asp^47^, Gln^50^, and Arg^55^ of CcmC and Arg^73^, Asp^101^, and Glu^105^ of CcmE are collectively essential for the successful association of the two proteins, leading to maturation of CcmE and cytochrome *c*.

### CcmC Gln^49^ interacts with CcmE Arg^104^, and this interaction ensures efficient holo-CcmE release from the CcmC–heme–CcmE complex

**Figure 3.**
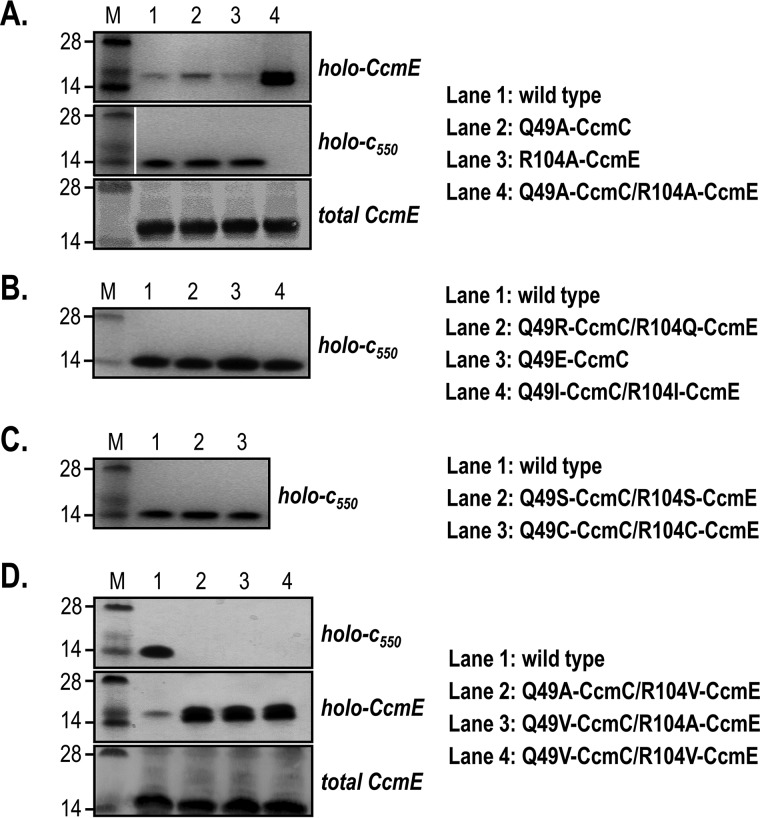
**The covarying residues, Gln^49^ and Arg^104^ in CcmC and CcmE, respectively, are involved in the release of holo-CcmE after covalent heme attachment.** *A*, single replacements of Gln^49^ and Arg^104^ by alanine do not affect holo-*c*_550_ levels, but a double variant leads to holo-CcmE accumulation and abrogation of cytochrome *c* maturation. *B*, swapping the positions of Gln^49^ and Arg^104^ on CcmC and CcmE or altering their polarity does not affect holo-*c*_550_ levels. *C*, replacement of Gln^49^ and Arg^104^ on CcmC and CcmE by serines or cysteines does not affect holo-*c*_550_ levels. *D*, replacement of the covarying pair by an alanine and a valine or two valine residues recapitulates the phenotype observed in *lane 4* of *A*. Levels of total CcmE are not affected by any of the mutations. Representative SDS-PAGE of cell membranes or periplasmic fractions stained for proteins containing covalently bound heme (*B* and *C* and *top* and *middle gels* of *A* and *D*) and immunoblotted to detect total CcmE amounts (*bottom gel* of *A* and *D*) are shown. Molecular weight markers (*M*) are on the *left*; the same marker lane was used for the *middle gel* of *A* and for *C. Gaps* indicate where a lane was removed.

After validating the CcmC–CcmE interaction site, we turned back to the residues identified by the covariance analysis (Gln^49^ in CcmC and Arg^104^ in CcmE) (42), whose role was still unclear. Our single-amino acid substitutions left holo-*c*_550_ amounts (*lanes 2* and *3* in Fig. 3*A*, *middle gel strip*) unaffected compared with the amounts generated by the WT System I (seen in *lane 1*). However, a double-alanine mutant led to a dramatic outcome; substantial accumulation of holo-CcmE was observed (*lane 4* in Fig. 3*A*, *top gel strip*), but holo-*c*_550_ production was entirely abrogated (*lane 4* in Fig. 3*A*, *middle gel strip*). Total CcmE amounts were assessed by immunoblotting for all constructs and were found to be unaffected (Fig. 3*A*, *bottom gel strip*).

The effect observed for the double-substitution is strongly reminiscent of the phenotype of the K40D-CcmA mutation, abolishing the ATPase activity of CcmA (26, 27). This suggests that CcmC Gln^49^ and CcmE Arg^104^ are likely playing an important role in the release of holo-CcmE from the CcmC–heme–CcmE complex. To further explore this hypothesis, we generated a series of System I variants and examined the amount of holo-*c*_550_ produced *in vivo*; in this experimental setting, WT levels of holo-*c*_550_ indicate that the substitution in question does not affect the CcmC–CcmE interaction and System I function. The mutations that were assessed are summarized in Table 1. We found that swapping the positions of the tested residues (Q49R-CcmC/R104Q-CcmE) or significantly altering their polarity (Q49E-CcmC and Q49I-CcmC/R104I-CcmE) had no effect (Fig. 3*B*). Several other mutations (Table 1), including double serine and cysteine substitutions (Fig. 3*C*), also did not affect System I function. We were only able to reproduce the result observed with the double-alanine substitution (Fig. 2*A*) when we replaced one or both alanines in this variant with a valine residue (Fig. 3*D*, *lanes 2–4*). For these mutants, we observed holo-CcmE accumulation and lack of holo-*c*_550_ production (*top* and *middle gel strips*), whereas total CcmE amounts remained unaffected (*bottom gel strip*).

**Table 1:** **Summary of results obtained by assessing the role of the covarying residues Gln^49^ and Arg^104^ in CcmC and CcmE, respectively (42), by mutagenesis on the entire *E. coli* System I operon and subsequent quantification of the relative amounts of holo-CcmE and exogenously expressed cytochrome *c*_550_ produced *in vivo*** For several variants, levels of holo-*c*_550_ were assessed first. WT amounts of *c*_550_ indicate that the mutation has no effect on the maturation process; in these cases, quantification of holo-CcmE was omitted (entries marked with — in the “Holo-CcmE level” column).

Variant	Holo-*c*_550_ level	Holo-CcmE level
Q49A-CcmC	WT level	Small increase
R104A-CcmE	WT level	WT level
Q49A-CcmC/R104A-CcmE	No holo-*c*_550_	Holo-CcmE accumulation
Q49R-CcmC/R104Q-CcmE	WT level	—
Q49E-CcmC	WT level	—
Q49I-CcmC/R104I-CcmE	WT level	—
Q49S-CcmC	WT level	—
R104S-CcmE	WT level	—
Q49S-CcmC/R104S-CcmE	WT level	—
Q49C-CcmC	WT level	—
R104C-CcmE	WT level	—
Q49C-CcmC/R104C-CcmE	WT level	—
Q49A-CcmC/R104V-CcmE	No holo-*c*_550_	Holo-CcmE accumulation
Q49V-CcmC/R104A-CcmE	No holo-*c*_550_	Holo-CcmE accumulation
Q49V-CcmC/R104V-CcmE	No holo-*c*_550_	Holo-CcmE accumulation
Q49K-CcmC/R104A-CcmE	WT level	—

Considering the outcome of our mutagenesis experiments (Table 1), the exact role of CcmC Gln^49^ and CcmE Arg^104^ is not clear. These residues are likely to interact, confirming the prediction by Ovchinnikov *et al.* (42). However, the interaction does not involve electrostatics or hydrogen bonding because replacement of both residues by isoleucine gave WT behavior (Table 1 and Fig. 3*B*). Surprisingly, double serine or cysteine substitutions (Fig. 3*C*), which are similar in side-chain volume to the double alanine or alanine/valine substitutions, did not elicit the phenotype observed in Fig. 3*A* (*lane 4*) and Fig. 3*D* (*lanes 2–4*) either, suggesting that side-chain size alone is not the determinant of the interaction. In the absence of a structure for CcmC, it is difficult to propose an exact function for the Gln^49^-CcmC-Arg^104^-CcmE pair. Nonetheless, we can suggest that these residues could play a steric role that would ensure that other residues in the CcmC–CcmE interaction surface are optimally placed for the release of holo-CcmE after heme attachment.

Bioinformatic analysis of the amino acids found at these positions in close homologues of *E. coli* CcmC and CcmE from bacteria and archaea (Table S2) shows that they can be occupied by a broad range of residues; however, the combinations that we identified as ones that are detrimental to System I function were not found to occur. We tested one of the alternative combinations that we identified bioinformatically for this pair (Q49K-CcmC/R104A-CcmE-System I variant) and found it to behave like the WT System I in terms of holo-*c*_550_ production (Table 1), confirming that several combinations of amino acids in these positions lead to a functional Ccm system.

### Holo-CcmE does not have a heme-binding site on the protein core

**Figure 4.**
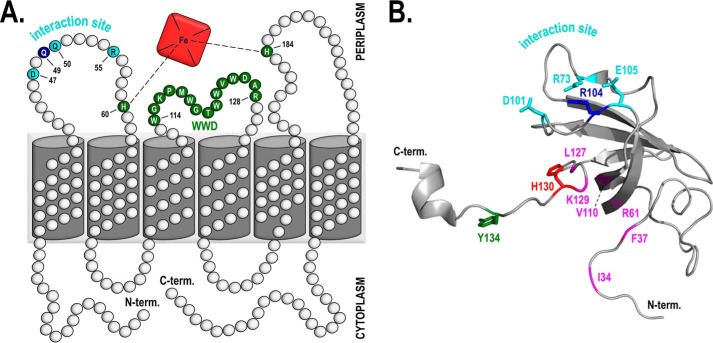
**The identified interaction site between CcmC and CcmE suggests that association of these two proteins is not driven by the presence of a heme-binding site on the CcmE protein core.** *A*, *E. coli* CcmC membrane topology model based on previously determined topologies (43). The WWD heme-binding motif and the heme axial ligands (His^60^ and His^184^) (22) are shown in *green*. The residues involved in the interaction with CcmE, identified in this study, are shown in *cyan*, and Gln^49^, which covaries with Arg^104^ in CcmE (42), is shown in *blue*. Heme is depicted by the *red square. B*, *schematic representation* of the three-dimensional structure of leaderless WT *E. coli* CcmE (PDB entry 1SR3) determined by NMR spectroscopy (37); only one of the 20 NMR structural conformers is displayed. Important residues are shown in a *stick representation*. His^130^ (*red*) binds heme covalently; Tyr^134^ (*green*) has been proposed to be an axial ligand of the covalently bound heme (49, 50) and has been found to be important for cytochrome *c* maturation (45, 48); Arg^73^, Asp^101^, and Glu^105^ (all in *cyan*) form the interaction site of CcmE with CcmC, identified in this study; and Arg^104^ (*blue*) covaries with Gln^49^ of CcmC (42). Residues shown in *magenta* (Ile^34^, Phe^37^, Arg^61^, Val^110^, Leu^127^, and Lys^129^) have been previously proposed to form a CcmE heme-binding site (37).

Our *in vivo* experiments showed that holo-CcmE formation depends on the association of CcmC with CcmE through six residues, three on each protein partner. This suggests that the interaction of CcmC with CcmE is independent of the heme moiety, as none of these System I residues have ever been found to be involved in heme binding. The location of these amino acids on CcmC and CcmE corroborates this assumption (Fig. 4). The structure of CcmC has not been determined; however, a reliable topological prediction has been proposed previously (43). Based on this topology model, on which we mapped the identified interaction residues and the covarying residue Gln^49^ (Fig. 4*A*), the CcmE interaction site does not coincide with the heme-binding site of CcmC, although it is sufficiently close for a productive subsequent interaction. The same can be concluded when the CcmE interacting residues are mapped on the 3D structure of the protein (Fig. 4*B*); these amino acids along with the covarying Arg^104^ form a tight cluster located on a flexible loop placed above His^130^, the *E. coli* CcmE heme-binding residue.

Nonetheless, the idea that CcmE has a heme-binding site and that its affinity for the heme moiety drives its association to heme-bound CcmC has been proposed numerous times. Attempts to validate this hypothesis have included a variety of techniques (37, 44–46), whereas residue-specific insight was provided by NMR spectroscopy and modeling (37). In the latter study, residues Ile^34^, Phe^37^, Arg^61^, and Val^110^, some of which are located in the protein β-barrel core, along with Leu^127^ and Lys^129^, which are part of its flexible C terminus (all shown in *magenta* in Fig. 4*B*), were postulated to form a heme-binding site. Some of these findings were validated *in vivo* in another study, using a minimal experimental system expressing only CcmC and CcmE (44). As these results are not consistent with our CcmC–CcmE interaction model, we employed NMR spectroscopy to re-examine the interaction of heme with soluble CcmE *in vitro* and to investigate whether a heme-binding site could be involved in its interaction with CcmC.

**Figure 5.**
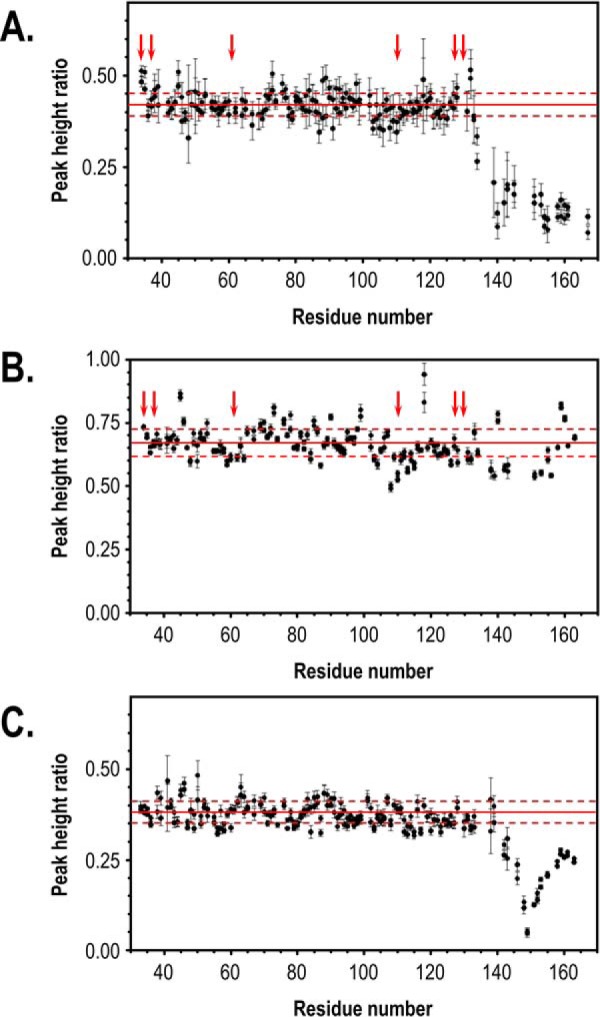
**Holo- and apo-CcmE do not have a heme-binding site on their protein core.** *A*, observed peak intensity ratios for ^15^N-labeled leaderless apo-CcmE and holo-CcmE bearing a C-terminal His_6_ tag, obtained from HSQC spectra collected at 750 MHz. Peak broadening, indicating interaction with the heme moiety, is only observed at the C terminus of the protein and only after residue 139. *B*, observed peak intensity ratios for ^15^N-labeled leaderless apo-CcmE and holo-CcmE without an affinity tag, obtained at 600 MHz. Almost no specific peak broadening is observed, showing that the heme has very few interactions with the protein backbone. *C*, observed peak intensity ratios for ^15^N-labeled leaderless H130A-apo-CcmE and H130A-holo-CcmE without an affinity tag, obtained at 750 MHz. Peak broadening is only observed at the C terminus of the protein, near residue 149. For all *panels*, the *solid red line* indicates the average peak ratio for residues 33–130, and the *dotted red lines* indicate the minimum and maximum values within one S.D. of this average. In *A* and *B*, the *red arrows* indicate the residues previously proposed to form a CcmE heme-binding site for holo-CcmE (37). Residues for which intensity ratios are not shown correspond either to prolines or to residues with overlapping or very weak peaks in the HSQC spectrum of apo-CcmE. For each residue, peak intensity ratios derived from two independent experiments are shown. Errors for each experiment (*error bars*) were derived from the Monte Carlo analysis of baseline noise.

Initially, we used a protein construct similar to that used by Enggist *et al.* (37) (*i.e.* leaderless (Ser^32^–Ser^163^) *E. coli* CcmE bearing a C-terminal His_6_ tag). However, instead of purifying the holoprotein after co-expression of soluble CcmE with System I, we purified it in its apo-form and fully reconstituted it with heme *in vitro*. The advantage of this approach is that we were certain that our sample contained solely WT holo-CcmE; we generally find that separation of mixtures of holo- and apo-CcmE produced *in vivo* is particularly challenging and never occurs fully. After staining our protein for covalently bound heme, we further characterized our sample by visible absorption spectra with and without hydroxide and pyridine in reducing heme conditions and found it to have the same heme attachment as in naturally occurring CcmE (Fig. S3*A*) (25). In addition, we used MS, which also confirmed that our sample only contained holo-CcmE. The heme in its oxidized state is paramagnetic, so we initially attempted to reduce the heme iron with sodium dithionite (Sigma-Aldrich), as claimed by Enggist *et al.* (37), before performing NMR experiments. However, we found that it was not possible to keep the protein in its reduced state, despite any measure taken against oxidation (degassed buffer system, argon flushing, etc.). We therefore proceeded to record ^1^H-^15^N heteronuclear single quantum coherence (HSQC) spectra of ^15^N-labeled apo-CcmE and ^15^N-labeled oxidized holo-CcmE (Fig. S3*B*) and assessed the effect of heme binding by comparing individual peak intensities for all backbone residues. Proximity of an amino acid with the paramagnetic heme should lead to peak broadening and decrease in the peak height (Fig. S3*C*); therefore, a lower peak intensity ratio should be observed for residues affected by the heme (Fig. 5*A*). We found that residues in the core of the protein showed a uniform level of nonspecific broadening that is to be expected because of the presence of the heme in the sample. Pronounced broadening was only observed at the C terminus, especially after residue 139. Whereas peaks for some of the residues cannot be observed due to very low intensity (*e.g.* His^130^, where the heme is bound) or cannot be included in the graph because of peak overlap (positions 129, 135, 141, 144, and 149), it is clear that the residues postulated by Enggist *et al.* (37) to form a heme-binding pocket do not show any specific broadening; Arg^61^ and Lys^129^ partially overlap with other residues, so they were not included in the graph, but the intensity of the overlapped peaks is also not affected more than other core residues by the presence of heme. We therefore conclude that the covalent attachment of the heme to this CcmE construct seems to be affecting the very end of the flexible C terminus, whereas there is no indication of a specific heme-binding site on the structured core of the protein.

The sample we examined (same construct as the protein used by Enggist *et al.* (37)), had a C-terminal His_6_ tag. Histidines are known to act as heme ligands, so to assess whether the broadening we observed at the C terminus of the protein (Fig. 5*A*) was due to the presence of the affinity tag, we also produced an *in vitro* heme-reconstituted CcmE sample devoid of the His_6_ tag. This was challenging because the His_6_ tag, although not essential, is useful for efficient heme attachment to the protein (46), but it poses significant problems at the cleavage step. By fusing a Streptavidin II tag after the His_6_ tag, we were able to produce a fully reconstituted holo-CcmE sample without an affinity tag, which we used to repeat the experiments described above. In this case, once more, residues in the core of the protein, including the ones postulated previously to form a heme-binding site, remained unaffected by heme. In addition, residues at the C terminus showed no significant broadening (Fig. 5*B*). This indicates that when heme is covalently bound to His^130^ in CcmE, it has very few specific interactions with the protein backbone.

Finally, we tested the overall flexibility of the protein backbone with and without attached heme using our initial His_6_-tagged samples by {^1^H}-^15^N heteronuclear NOE experiments (an example for apo-CcmE is shown in Fig. S4). For both samples, residues in the β-sheet structure generally gave values higher than 0.75, indicating a rigid backbone. Residues with NOE values lower than 0.75 are either located at the N terminus, which is expected as this protein is linked to a transmembrane helix in the cell, or at the C terminus. At the C terminus, residue 128 is the first amino acid with a reduced NOE value (0.68), and although we cannot measure values for residues 129 and 130, Asp^131^ has a significantly lower NOE ratio of 0.49, suggesting that the protein is likely flexible from residue 128 onward. The fact that the results were similar for both apo- and holo-samples also suggests that the presence of the attached heme does not alter the flexibility of the protein.

### Apo-CcmE does not have a heme-binding site on the structured protein core

We found that there is no heme-binding site on the structured core of holo-CcmE. However, this species occurs after covalent heme attachment and could well not require any further interaction with the heme moiety. The most obvious way to assess the noncovalent interaction of apo-CcmE with heme would be to perform NMR experiments on a mixture of WT apo-CcmE and heme, soon after the heme addition. Unfortunately, we found that for protein concentrations required for NMR spectroscopy (minimum of 100 μm), the addition of heme led to rapid (<10-min) covalent heme attachment to the protein (Fig. S5, *lane 1*). This occurred even when we used WT apo-CcmE devoid of its C-terminal His_6_ tag (Fig. S5, *lane 2*), which has been shown previously to be slower in covalently attaching heme *in vitro* (46). For this reason, we generated a sample that mimics CcmE before heme attachment. We used H130A-CcmE, which lacks its heme-binding histidine residue and therefore is unable to bind heme covalently; this particular CcmE variant has been used successfully in previous studies as a mimic of the pre-adduct state of CcmE (35, 47). After purification of the ^15^N-labeled apoprotein, we cleaved the His_6_ tag to avoid any interference with heme binding, and we added an excess of heme. After a short incubation, we removed the excess heme through a rigorous wash process. The resulting sample, shown in Fig. S6, contains heme bound to the protein noncovalently and can be used to assess whether apo-CcmE has a specific heme-binding pocket before covalent heme attachment. We repeated the same ^1^H-^15^N HSQC NMR experiments we performed for WT holo-CcmE (Fig. 5, *A* and *B*), and once more we found that there is no specific broadening in the structured core of the protein that would indicate a heme-binding site (Fig. 5*C*). We note that comparison of these results with the graph obtained for cleaved WT holo-CcmE (Fig. 5*B*), shows that there is more specific broadening at the C terminus of H130A-CcmE. Nonetheless, most of the broadening is around Arg^149^, and very little broadening is observed for residues 130–133, which are the site of covalent heme attachment in the WT protein.

## Discussion

The purification of the complex between heme-bound CcmC and CcmE (22, 24), provided very important new insights into the heme trafficking processes of System I. These studies made clear that CcmE can attach heme covalently only when in complex with CcmC and while the heme is ligated by the essential CcmC histidines (His^60^ and His^184^) (24). However, the mechanistic drivers of the interaction between heme-bound CcmC and apo-CcmE remained elusive until now, especially because the 3D structure of CcmC is still lacking. The use of advanced *in silico* analyses was key for identifying potential interacting residues between these two proteins. The work by Ovchinnikov *et al.* (42) pinpointed in which region of each protein we should search for interacting residues, and by combining bioinformatics with *in vivo* experiments, we were able to identify a protein–protein interaction site between CcmC and CcmE consisting of six residues (Asp^47^, Gln^50^, and Arg^55^ on CcmC; Arg^73^, Asp^101^, and Glu^105^ on CcmE). We found that replacement of each of these amino acids with alanine led to significant decrease in holo-CcmE and holocytochrome *c* formation (Fig. 2, *A* and *B*), whereas the absence of all three residues on one of the interacting partners abolished System I function entirely (Fig. 2*C*). The latter proves that each of the identified residues contributes to, and that they are collectively essential for, the interaction of CcmC with CcmE. The fact that production of holocytochrome *c* mirrored holo-CcmE maturation for each of the variants (Fig. 2, *A* and *B*), further demonstrates that these residues affect the association rather than the dissociation of these two proteins. In addition, because single-amino acid substitutions on either side of the interacting surface (CcmC or CcmE) have the same effect (Fig. 2, *A* and *B*), these six residues are potentially organized into three interacting pairs. Based on the nature of the identified amino acids, one of these interacting pairs likely involves Asp^47^ in CcmC and Arg^73^ in CcmE.

**Figure 6.**
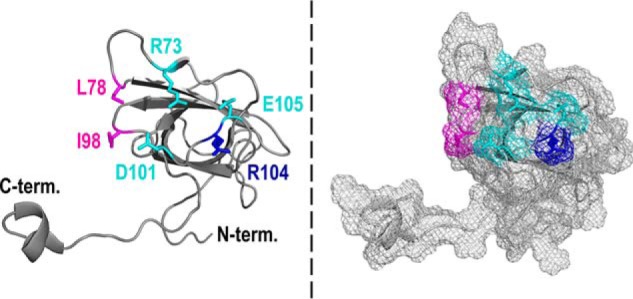
**Arg^73^, Asp^101^, Glu^105^ (in *cyan*), and Arg^104^ (in *blue*) form a relatively large interaction interface on the periplasmic domain of CcmE.** Only two additional residues near the identified interaction site of CcmE, Leu^78^ and Ile^98^ (in *magenta*), are sufficiently surface-exposed and have side chains with the right orientation to potentially be part of this interaction interface. Shown is a *schematic representation* of the 3D structure of leaderless WT *E. coli* CcmE (PDB entry 1SR3) determined by NMR spectroscopy (37) (*left*, *cartoon representation* of the backbone and *stick representation* of residues of interest; *right*, *space-filled representation* in the same orientation).

Visualization of the three identified interacting residues (Arg^73^, Asp^101^, and Glu^105^) on the structure of CcmE (Fig. 6) reveals a relatively large interaction interface for the periplasmic domain of this protein. Although we cannot exclude the contribution of other residues to this interaction interface, careful examination of the CcmE structure showed that only two additional residues, Leu^78^ and Ile^98^ (Fig. 6, *magenta*), are sufficiently surface-exposed and have side chains with the right orientation to be part of it. These are nonpolar residues. Therefore, if indeed they were involved in the interaction of CcmE with CcmC, they would most likely contribute weaker van der Waals interactions with their partners on CcmC. In the absence of a structure for the latter, it is not possible to venture a guess regarding which CcmC residues Leu^78^ and Ile^98^ of CcmE could be interacting with. We also note here that the six residues we identified are likely not the sole determinant of the overall CcmC–CcmE interaction. Whereas they are certainly extremely important for the association of the periplasmic domain of apo-CcmE with the periplasmic portion of heme-bound CcmC, as confirmed by the dramatic phenotypes observed by their substitution (Fig. 2), the proteins of System I are thought to generally associate with each other, forming either one large supercomplex or several smaller complexes (21, 35). These interactions are no doubt also driven by the membrane-embedded domains of these proteins, although exact mechanistic evidence is not yet available.

When it came to the pair of covarying residues, CcmC Gln^49^ and CcmE Arg^104^, their role was not immediately obvious. We performed a series of mutagenesis experiments, which did not affect any of the System I functions that we were able to assess (Table 1). It was only when both residues were replaced by nonpolar amino acids that a phenotype was observed (Fig. 3, *A* and *D*). Replacement of these residues with alanines or valines led to impressive accumulation of holo-CcmE and the complete absence of holocytochrome *c* formation, strongly reminiscent of the K40D-CcmA-System I phenotype (26, 27). This suggests that although holo-CcmE successfully formed, it could not be released from the CcmC–heme–CcmE complex, despite the fact that CcmAB were present and functional. As the covarying residues are located within the CcmC–CcmE interaction site, a plausible explanation for this phenotype is that they also interact, as predicted by Ovchinnikov *et al.* (42), and their role is a steric one. More specifically, their presence would ensure that during CcmC–CcmE association, these two proteins do not interact too tightly; otherwise, the action of CcmAB would not be sufficient to allow release of holo-CcmE. This hypothesis is supported by our bioinformatics data (Table S2), which show that although there is a significant variation in the amino acids found at these positions in related bacterial species, the combinations leading to the phenotype we observed (A-A, A-V, V-A, and V-V) are never found. The proposed steric role for these residues makes sense if one considers that although CcmC and CcmE interact with high affinity (22, 24), the CcmC–heme–CcmE complex has to be eventually resolved for cytochrome *c* to be matured.

The question of whether CcmE interacts with heme-bound CcmC through a heme–protein interaction mediated by a heme-binding site on CcmE has been a longstanding one. On the one hand, this assumption is a logical one for a “heme chaperone”; hence, the attempts to detect such an interaction site (37, 44–46) are justified. On the other hand, none of the aforementioned studies produced convincing data that proved the presence of such a site. We found that apo- and holo-CcmE do not have a heme-binding site in the core of the protein (Fig. 5). WT holo-CcmE shows some specific broadening at the very end of its C terminus when it bears a His_6_ tag and no interaction with its attached heme when cleaved of its affinity tag. The protein core of His^130^-holo-CcmE, which we use as a mimic of the protein before covalent bond formation, was also not found to interact with the heme moiety. Although there is some broadening at the C terminus, there is no interaction of the heme with the structured part of CcmE, and certainly there is no detectable CcmE heme-binding pocket on the main body of the protein. The results of our NMR analysis agree with the findings of our *in vivo* experiments, where we showed that association of CcmC with CcmE occurs through protein–protein rather than protein–heme interactions and are not surprising if one interprets them with the *in vivo* function of CcmE in mind. For the pre-adduct species, it is important to consider that this protein attaches heme covalently while already in association with heme-bound CcmC in a stable complex and while the heme cofactor is ligated to two histidine residues on CcmC (24). For holo-CcmE, the lack of a heme-binding site is also unsurprising. *In vivo* this species is binding the heme stably through its unique covalent bond and needs to pass it on to the substrate apocytochrome. In this context, a heme-binding site on the structured core of the periplasmic domain of CcmE would likely be detrimental to the overall role of this protein, as it would not facilitate the transfer step of the heme to the apocytochrome.

### CcmC–CcmE interaction model

**Figure 7.**
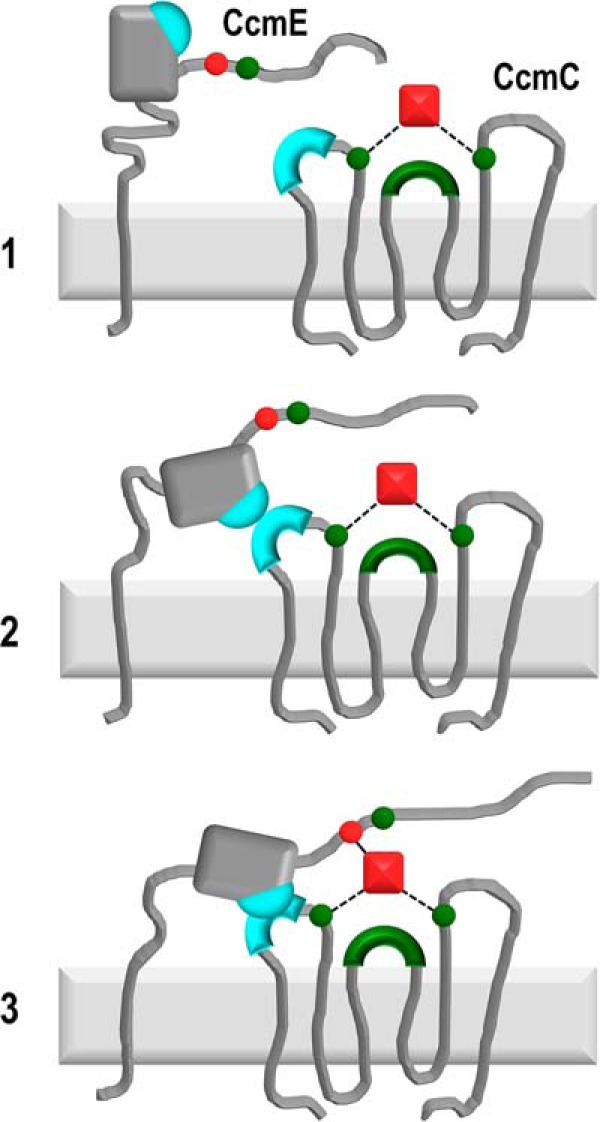
***Schematic representation* of the proposed mechanism for the interaction of CcmE with heme-bound CcmC and for the subsequent covalent heme attachment to CcmE.** Heme is depicted as a *red square*, and CcmE His^130^, which binds heme covalently, is shown as a *red circle*. Residues that ligate heme (CcmE Tyr^134^; CcmC His^60^ and His^184^) or bind heme noncovalently (CcmC WWD motif) are shown in *green*. The interaction site between CcmC and CcmE is marked in *cyan* on both proteins. Covalent bonds involving the heme moiety are displayed by a *solid line*, and heme ligation is shown by *dotted lines*. The sequence of events is proposed to be as follows. *1*, CcmC ligates heme through two histidine residues (His^60^ and His^184^) flanking the WWD motif (*green circles* and *arch*) (22). *2*, heme-bound CcmC associates with apo-CcmE through three pairs of residues, Asp^47^, Gln^50^, and Arg^55^ located on the first periplasmic loop of CcmC and Arg^73^, Asp^101^, and Glu^105^ located on a loop of the β-barrel core of CcmE (see also Fig. 4), forming a stable CcmC–heme–CcmE complex (22, 24). During this step, Gln^49^ of CcmC and Arg^104^ of CcmE (covarying pair) play a steric role, ensuring that the association of the two proteins is optimal. *3*, after the CcmC–heme–CcmE complex is formed, the flexible C-terminal tail of CcmE is positioned near the heme, likely through interaction with Tyr^134^ (45, 48–50), and this environment of high proximity leads to covalent attachment of heme to His^130^ of CcmE. As CcmD is not found to be essential for formation of holo-CcmE in the context of the CcmC–heme–CcmE complex (41), it has been omitted from this model.

Using covariance and bioinformatics analyses, *in vivo* experiments on the entire System I operon, and NMR spectroscopy, we can propose for the first time a mechanistic model (Fig. 7) for the interaction of CcmC with CcmE leading to holo-CcmE formation. We suggest the following sequence of events. CcmC sources the heme for System I and binds it through its WWD motif and through ligation via two conserved histidine residues, His^60^ and His^184^ in *E. coli* (22, 24) (Fig. 7, *step 1*). CcmE associates with heme-bound CcmC via three pairs of interacting residues (Asp^47^, Gln^50^, and Arg^55^ on CcmC; Arg^73^, Asp^101^, and Glu^105^ on CcmE) forming a stable CcmC–heme–CcmE complex. During this association, Gln^49^ and Arg^104^ in CcmC and CcmE, respectively, also interact, exerting a steric role within the CcmC–CcmE interaction interface that prevents the two proteins from binding too tightly to each other and makes the subsequent release of holo-CcmE possible (Fig. 7, *step 2*). In the CcmC–heme–CcmE complex environment, the CcmC–CcmE interaction places CcmE in an optimal position for covalent heme attachment. Its flexible tail is brought into proximity of the heme moiety by the interaction interface and is likely further oriented toward the heme by Tyr^134^ (Fig. 7, *step 3*). Tyr^134^ is almost entirely conserved (in ∼3% of cases, it is a Phe, also a plausible heme ligand), and, although not essential, it has been shown to be important for efficient holo-CcmE production (45, 48) and has been proposed to be the heme ligand after holo-CcmE release (49, 50). It can be envisaged that the proximity of His^130^ to the heme at this point would be sufficient for covalent bond formation through a Michael addition or an imidazole cation radical mechanism (19).

In the absence of high-resolution structures for most of the System I components, studies like this contribute significantly to our understanding of the mechanistic details of this multiprotein system. Our model (Fig. 7) provides important new insight into one of the crucial steps of cytochrome *c* biogenesis as performed by System I, which not only operates in Gram-negative bacteria but is also found in the mitochondria of most photosynthetic eukaryotes. In addition, our results demonstrate the great potential of *in silico* predictions for the study of complex protein–protein interactions. A similar approach could be followed to elaborate further the interaction of mitochondrial apo-cytochrome *c* with its maturation apparatus in most nonphotosynthetic eukaryotes, a single protein called holocytochrome *c* synthase (HCCS). Despite important recent progress in this research direction (51), the structure of HCCS remains unknown, and therefore gaining residue-specific insight in the maturation process is challenging. Use of covariance and bioinformatics methods could reveal which parts of HCCS are involved in the interaction with its substrate apocytochrome.

## Experimental procedures

### Construction of plasmids

Plasmids and oligonucleotides used in this study are listed in Tables S3 and S4, respectively. All constructs, with the exception of pQE2-Im9 and pCcmC1, were produced by site-directed mutagenesis (QuikChange, Stratagene) using plasmid pE221 (52) or pEC86 (53) as template. pQE2-Im9 was generated by amplifying the gene of the immunity protein of colicin E9 (immunity 9) from *E. coli* genomic DNA using oligonucleotides P1 and P2 and ligating it into AseI-SphI–digested TAGZyme pQE vector (pQE-2, Qiagen). The resulting expression vector encodes an N-terminal polyhistidine tag, followed by the immunity 9 gene and a Factor Xa cleavage site before the multiple cloning site of TAGZyme pQE. pCcmC1 was generated by amplifying *E. coli ccmC* from pEC86 using oligonucleotides P3 and P4 and ligating it into NdeI-HindIII–digested pQE2-Im9 (Table S3). DNA manipulations were conducted using standard methods. Oligonucleotides were purchased from Sigma Genosys. KOD Hot Start DNA polymerase (Novagen) was used for all PCRs, enzymes were purchased from New England Biolabs, and all constructs were sequenced before use.

### Cell growth and fractionation

Bacterial strains used in this study are listed in Table S1. Cell growth and fractionation for *in vivo* experiments were carried out as follows. Experiments were performed in the WT *E. coli* strain JCB38725 (54). The *E. coli ccm* operon or its variants were constitutively expressed from plasmid pEC86 or its derivative plasmids generated in this study, respectively (Table S3). *Paracoccus denitrificans* cytochrome *c*_550_ was co-expressed with the *E. coli ccm* operon (or its variants) from plasmid pKPD1 (55). *E. coli ccmC* or its variants were expressed from plasmids pCcmC1 or pSHS47–49, respectively (Table S3), specifically for assessing total CcmC amounts. In all cases, cell growth was conducted in 200 ml of 2×TY medium (16 g/liter peptone, 10 g/liter yeast extract, and 5 g/liter NaCl) in 2.5-liter flasks; the small culture volumes ensured sufficient aeration, preventing expression of the endogenous *E. coli ccm* genes. Cultures were inoculated from single colonies and incubated at 37 °C for 15–18 h with shaking at 200 rpm. 1 mm isopropyl-1-thio-β-d-galactopyranoside was added to the cultures from inoculation. 100 μg/ml ampicillin and 34 μg/ml chloramphenicol were used when appropriate. For the extraction of periplasmic fractions, cells were harvested and spheroplasted as described by Allen *et al.* (56). For the isolation of the crude membrane fractions, a French press was used. Disruption of the cells was performed at 16,000 p.s.i., followed by centrifugation at 257,000 × *g* for 1 h at 4 °C. The membrane fraction was resuspended in ∼25 ml of 50 mm Tris-HCl (pH 7.5), 150 mm NaCl and was centrifuged again as above. The washed crude membrane fraction was resuspended in 1–2 ml of 50 mm Tris-HCl (pH 7.5), 150 mm NaCl.

Cell growth and fractionation for protein production and purification were carried out as follows. *E. coli* BL21 (DE3) (Stratagene) were transformed with plasmid pE221 (52) or its derivative plasmids generated in this study (pSHS04, pSHS05, and pSHS10; Table S3) expressing leaderless (Ser^32^–Ser^163^) *E. coli* CcmE. Transformants were initially grown on lysogeny broth (LB; 10 g/liter peptone, 5 g/liter yeast extract, 10 g/liter NaCl) agar plates supplemented with 100 μg/ml ampicillin. For production of unlabeled protein samples, transformants were subsequently used to inoculate small-scale overnight cultures (5 ml of LB) in 50-ml polypropylene tubes, supplemented with 100 μg/ml ampicillin, and incubated at 37 °C with shaking at 200 rpm for 15–18 h. 500 ml of LB in 2.5-liter flasks were then inoculated from the overnight cultures (1:250), and the resulting suspensions were incubated at 37 °C with shaking at 200 rpm until *A*_600_ = 0.8. 1 mm isopropyl-1-thio-β-d-galactopyranoside was used for protein induction, and cultures were further grown at 30 °C with shaking at 200 rpm for 15 h. For the production of ^15^N-labeled protein samples, cell growth and protein induction were carried out as described in previous work (57). For all cultures, after harvesting, the periplasmic fraction was isolated as described above.

### Apo-CcmE purification and in vitro reconstitution with heme

Unlabeled or ^15^N-labeled WT or H130A leaderless *E. coli* apo-CcmE was purified as follows. Cells were grown as described above, and, after isolation, the periplasmic fraction was applied onto 10 ml of Fast Flow Chelating Sepharose (Amersham Biosciences) charged with Ni^2+^ and equilibrated with 50 mm Tris-HCl (pH 7.5), 300 mm NaCl. The column was washed with the equilibration buffer, and apo-CcmE was eluted with 50 mm Tris-HCl (pH 7.5), 300 mm NaCl, 200 mm imidazole. The eluent was exchanged into 50 mm Tris-HCl (pH 7.5), 150 mm NaCl and concentrated. Thrombin cleavage of the affinity tag, when necessary, was performed using the Thrombin CleanCleave kit (Sigma) according to the manufacturer's instructions.

WT holo-CcmE bearing a C-terminal His_6_ tag was generated by *in vitro* reconstitution of the purified apoprotein (from plasmid pE221; Table S3) with heme, as follows. A 5-fold excess of hemin (Sigma-Aldrich; dissolved in DMSO) was added into a 1 mm protein solution along with 2 mm disodium dithionite. The reactions were carried out in the dark and at room temperature under humidified argon gas for 16–18 h. Unbound heme was removed by incubating the reaction mixtures in the dark with equal volumes of 50 mm Tris-HCl (pH 7.5), 150 mm NaCl, 2 m imidazole for 16–18 h at 4 °C. Excess imidazole and unbound heme were removed by applying the reaction mixture to a desalting column (0.6 × 15 cm) packed with hydrated BIO-Gel P-6 DG resin (Bio-Rad).

WT holo-CcmE without an affinity tag was generated through a multistep *in vitro* heme reconstitution process. Whereas the presence of the His_6_ tag is important for efficient and timely covalent heme attachment, it causes significant protein loss at the cleavage step. Thus, a construct bearing a C-terminal His_6_ tag preceded by a thrombin cleavage site and followed by a Strep-II tag was used (pSHS05; Table S3). The apoprotein was produced as described above and purified through its C-terminal His_6_ tag. Subsequently, covalent heme attachment and thrombin cleavage were performed as described above. After the cleavage reaction, the cleaved affinity tag was removed by applying the reaction mixture onto 5 ml of Strep-Tactin Sepharose (IBA), equilibrated with 50 mm Tris-HCl (pH 7.5), 150 mm NaCl. The flow-through was collected, and the resin was further washed with the equilibration buffer. Although this step led to ∼60–70% protein loss, due to association of the heme of holo-CcmE with the His_6_ tag that was bound on the affinity column, a sufficient amount of cleaved protein for NMR spectroscopy was recovered.

H130A-holo-CcmE devoid of a His_6_ tag was generated by *in vitro* reconstitution of the purified apoprotein (from plasmid pSHS10; Table S3) with heme, as follows. H130A-apo-CcmE cleaved of its C-terminal His_6_ tag was mixed with excess heme (Sigma-Aldrich; dissolved in DMSO) and incubated at room temperature for ∼30 min. The excess heme and DMSO were subsequently removed by buffer exchange in a concentrating device using 50 mm Tris-HCl (pH 7.5), 150 mm NaCl.

### Characterization of pure protein samples

Protein concentration was determined using the Pierce BCA Reducing Agent Compatible Protein Assay Kit (Thermo Fisher Scientific). All purified proteins were subjected to SDS-PAGE analysis and electrospray ionization MS (ESI-MS) to confirm that they were pure and of the expected masses. SDS-PAGE analysis was carried out on 10% BisTris NuPAGE gels (Thermo Fisher Scientific) with prestained molecular weight markers (SeeBlue Plus 2; Thermo Fisher Scientific). All samples were denatured at 100 °C for 2 min. Simply Blue Safe stain (Thermo Fisher Scientific) was used for staining gels for total protein content. For detection of proteins with covalently bound heme, the method of Goodhew *et al.* (58) was used; for WT holo-CcmE protein solutions, this method was used to initially confirm that the reconstitution of apo-CcmE with heme was successful. ESI-MS was performed using a Micromass Bio-Q II-ZS triple quadrupole mass spectrometer (10-μl protein samples in 1:1 water/acetonitrile, 1% (v/v) formic acid at a concentration of 20 pmol/μl were injected into the electrospray source at a flow rate of 10 μl/min). For WT holo-CcmE protein solutions, ESI-MS was used as a definitive method to assess whether the protein was fully reconstituted with heme.

Apo- and holo-WT CcmE solutions were found to be folded as they gave well dispersed NMR spectra (Fig. S3*B*; details for acquisition of spectra are given below). The backbone chemical shifts of WT apo-CcmE recorded by 2D ^1^H-^15^N HSQC spectroscopy are in agreement with the published chemical shifts used for structural determination of apo-CcmE (37). The spectrum of the holoprotein is largely similar to that of apo-CcmE, showing that covalent heme attachment does not affect its overall fold.

The protocol for *in vitro* reconstitution of apo-CcmE with heme has been shown previously to produce fully functional protein able to transfer its heme to an apocytochrome (52). Nonetheless, and in addition to MS, which confirmed the correct masses for all apo- and holo-samples, holo-CcmE solutions were also assessed by visible absorption spectra on a Varian Cary 50 Bio spectrophotometer (Fig. S3*A*). Sample reduction was performed by the addition of several grains of sodium dithionite. In addition, absorption spectra in the presence of hydroxide and pyridine in reducing heme conditions were obtained (Fig. S3*A*, *inset*) according to the method of Bartsch (59). The latter are characteristic of the type of iron-porphyrin present as well as of any modifications to it. For all *in vitro*-reconstituted WT holo-CcmE samples, the same heme attachment as in naturally occurring CcmE (25) was observed.

### Characterization of periplasmic and membrane fractions

Production of cytochrome *c*_550_ was assessed by SDS-PAGE analysis of periplasmic fractions, followed by detection of proteins with covalently bound heme. Periplasmic extraction (56), SDS-PAGE analysis, and heme staining (58) were performed as described above. Gel loadings were normalized according to wet cell pellet weights, and samples were denatured at 100 °C for 2 min. At least six replicates of each experiment were conducted.

Production of holo-CcmE was assessed by SDS-PAGE analysis of membrane fractions, followed by detection of proteins with covalently bound heme. Isolation of the crude membrane fractions, SDS-PAGE analysis, and heme staining (58) were performed as described above. Gel loadings were normalized according to the total protein content of the membrane suspensions, determined using the Pierce BCA Reducing Agent Compatible Protein Assay Kit (Thermo Fisher Scientific), and samples were denatured by incubation at 42 °C for 5 min. At least three replicates of each experiment were conducted.

Immunoblotting was carried out following SDS-PAGE analysis by transferring the proteins onto nitrocellulose (Hybond C-Extra, Amersham Biosciences). Blocking was performed with 5% (w/v) skimmed milk powder in TBS (50 mm Tris-HCl (pH 7.5), 120 mm NaCl, 0.1% (v/v) Tween 20). For the detection of total CcmE amounts in crude membrane fractions, rabbit antiserum raised against *E. coli* CcmE (Covalab) (dilution 1:1000) was used, followed by mouse anti-rabbit alkaline phosphatase–conjugated antibody (Sigma) (dilution 1:30,000). For the detection of total CcmC amounts in crude membrane fractions, a penta-His horseradish peroxidase alkaline phosphatase–conjugated mAb (Sigma) (dilution 1:2000) was used. Development was carried out using a SigmaFast 5-bromo-4-chloro-3-indolyl phosphate/nitro blue tetrazolium tablet (Sigma) in both cases. At least three replicates of each experiment were conducted.

### NMR spectroscopy

NMR experiments were carried out using spectrometers operating at ^1^H frequencies of 600 and 750 MHz. The spectrometers were equipped with Oxford Instruments magnets and home-built triple-resonance pulsed-field gradient probes (750 MHz) or with Bruker Avance consoles and TCI CryoProbes (600 and 750 MHz). NMR data were acquired using either GE/Omega software with pulse sequences written in-house or Topspin software and pulse sequences provided in the Topspin libraries from Bruker Biospin. NMR data were processed using NMRPipe (60), and spectra were analyzed using the CCPN software (61). Protein samples were prepared at concentrations of ∼0.3–1.2 mm in 25 mm Tris (95:5, H_2_O/D_2_O), at pH values ranging from 5.5 to 7.5. All experiments were conducted at 25 °C.

2D ^1^H-^15^N HSQC spectra were recorded at 600 MHz with spectral widths of 7,575.76 and 1,901.14 Hz in the ^1^H and ^1^5N dimensions, respectively, or at 750 MHz with spectral widths of 9,433.96 and 2,380.95 Hz in the ^1^H and ^15^N dimensions, respectively. HSQC data sets were collected with 128 and 1024 complex points in F_1_ (^15^N) and F_2_ (^1^H), respectively. The peak height ratios shown in Fig. 5 were calculated as the ratio of the peak intensities in the spectra recorded for the holo- and apoproteins. Peak heights were determined using the CCPN Analysis software (61). Uncertainties in the peak height ratios were estimated from 500 Monte Carlo simulations using the baseline noise as a measure of the error in the peak heights. Two replicates of each experiment were collected and are plotted in Fig. 5.

The {^1^H}-^15^N heteronuclear NOE experiments were recorded with and without ^1^H saturation for 4 s at 750 MHz in an interleaved manner. The data sets were acquired using the same sweep widths and numbers of data points as for the HSQC experiments. The {^1^H}-^15^N NOE was calculated as the ratio of the peak intensities in the spectra recorded with and without ^1^H saturation. Peak heights and errors in the {^1^H}-^15^N NOE were determined as described above.

### Resonance assignments

Resonance assignments for *E. coli* CcmE have been reported previously at pH 7.2 in 50 mm sodium phosphate, 300 mm NaCl at 30 °C (BMRB accession number 6723) (37). These assignments were used as the starting point for assignment of the HSQC spectrum of WT apo-CcmE (with and without a C-terminal His_6_ tag) and H130A-apo-CcmE (without a C-terminal His_6_ tag) at 25 °C at pH values ranging from 5.5 to 7.5. Assignments under our solution conditions were confirmed using 3D ^15^N-edited total correlation spectroscopy-HSQC and NOESY-HSQC data sets.

### Bioinformatics

To assess the conservation of *E. coli* CcmC or CcmE residues of interest, 2,753 bacterial and archaeal representative proteomes from the NCBI database were searched for homologs of *E. coli* CcmC or CcmE, respectively, using blastp 2.2.28+ (NCBI) (62) (*e* value <0.01, identity >20%, coverage >50%). Hits were used as a blastp query against the *E. coli* MG1655 proteome (*e* value <0.01), and only the ones giving CcmC or CcmE as best-hit results were retained. The sequences were aligned using MUSCLE version 3.8.31 (63) and checked manually for consistency. The retained sequences were clustered using ucluster version 7.0 (identity = 0.2) (64), and representative sequences of the main clusters were selected and aligned along with the *E. coli* MG1655 CcmC or CcmE homolog using MUSCLE 3.8.31.

To identify amino acids found in positions corresponding to Gln^49^ and Arg^104^ of *E. coli* CcmC and CcmE, respectively, in other Gram-negative bacteria, the same search procedure was used using blastp with *e* value <1E−5, identity >40%, and coverage >80%. A total of 628 were retrieved. Sequences were aligned using MUSCLE 3.8.31 (63), and residues in positions corresponding to Gln^49^ and Arg^104^ in the *E. coli* MG1655 CcmC and CcmE homologs, respectively, were retrieved. The frequency of occurrence of each pair was subsequently calculated on the subset of sequences from α- and γ-proteobacteria to avoid confounding effects due to excessive divergence.

## Author contributions

S. J. F., C. R., and D. A. I. M. designed research; S. H. S., D. G., and C. R. performed research; D. G., S. J. F., C. R., and D. A. I. M. analyzed the data; J. L. C. and C. K provided materials; C. R. and D. A. I. M. wrote the paper with contribution from the other authors.

## Supplementary Material

Supporting Information

## Footnotes

4 The abbreviations used are:

Ccm: cytochrome *c* maturation
holo-*c*_550_: holocytochrome *c*_550_
HSQC: heteronuclear single quantum coherence
HCCS: holocytochrome *c* synthase
immunity 9: immunity protein of colicinE9
LB: lysogeny broth
ESI: electrospray ionization
2D and 3D: two- and three-dimensional, respectively
BisTris: 2-[bis(2-hydroxyethyl)amino]-2-(hydroxymethyl)propane-1,3-diol.
