# Supplementary material for: The CcmC–CcmE interaction during cytochrome c maturation by System I is driven by protein–protein and not protein–heme contacts
Source: J Biol Chem. 2018 Sep 11;293(43):16778–90. doi: 10.1074/jbc.RA118.005024 (PMC6204919; doi:10.1074/jbc.RA118.005024)
Supplement: Supporting Information [file supp_293_43_16778__index.html]

The CcmC-CcmE interaction during cytochrome c maturation by System I is driven by protein-protein and not protein-heme contacts — Mapping the CcmC-CcmE interaction in System I — The CcmC–CcmE interaction during cytochrome c maturation by System I is driven by protein–protein and not protein–heme contacts — Mapping the CcmC–CcmE interaction in System I — Supporting Information 

# The CcmC–CcmE interaction during cytochrome *c* maturation by System I is driven by protein–protein and not protein–heme contacts

## Supporting Information

- Supporting Information (to be published online) - PDF including Fig. S1-S6, Tables S1-S4 and Supporting Information reference citations
