# Supplementary material for: The CcmC–CcmE interaction during cytochrome c maturation by System I is driven by protein–protein and not protein–heme contacts
Source: J Biol Chem. 2018 Sep 11;293(43):16778–90. doi: 10.1074/jbc.RA118.005024 (PMC6204919; doi:10.1074/jbc.RA118.005024)
Supplement: Supporting Information [file supp_RA118.005024_139624_1_supp_198928_p8p68y.pdf]

SUPPORTING INFORMATION

The CcmC-CcmE interaction during cytochrome *c* maturation by System I is driven by protein-protein and not protein-heme contacts

Shevket H. Shevket[2], Diego Gonzalez[3], Jared Cartwright[4], Colin Kleanthous[2], Stuart J. Ferguson[2,*], Christina Redfield[2,*], and Despoina A.I. Mavridou[1,*]

From the [1]MRC Centre for Molecular Bacteriology and Infection, Department of Life Sciences, Imperial College London, Kensington, London, SW7 2DD, UK; [2]Department of Biochemistry, University of Oxford, South Parks Road, Oxford, OX1 3QU, UK; [3]Department of Fundamental Microbiology, Faculty of Biology and Medicine, University of Lausanne, Quartier UNIL/Sorge, Lausanne, Switzerland, [4]Bioscience Technology Facility, Department of Biology, University of York, Wentworth Way, York, YO10 5DD, UK.

[*]To whom correspondence should be addressed: Despoina A.I. Mavridou: MRC Centre for Molecular Bacteriology and Infection, Department of Life Sciences, Imperial College London, Kensington, London, SW7 2DD, UK; d.mavridou@imperial.ac.uk; Tel. +44(0)2075949936; Christina Redfield: Department of Biochemistry, University of Oxford, South Parks Road, Oxford, OX1 3QU, UK; christina.redfield@bioch.ox.ac.uk; Tel. +44(0)1865275330; Stuart J. Ferguson: Department of Biochemistry, University of Oxford, South Parks Road, Oxford, OX1 3QU, UK; stuart.ferguson@bioch.ox.ac.uk; Tel. +44(0)1865613299.

This PDF file includes:

Figures S1 to S6
Tables S1 to S4
Supporting Information reference citations

SUPPORTING FIGURES

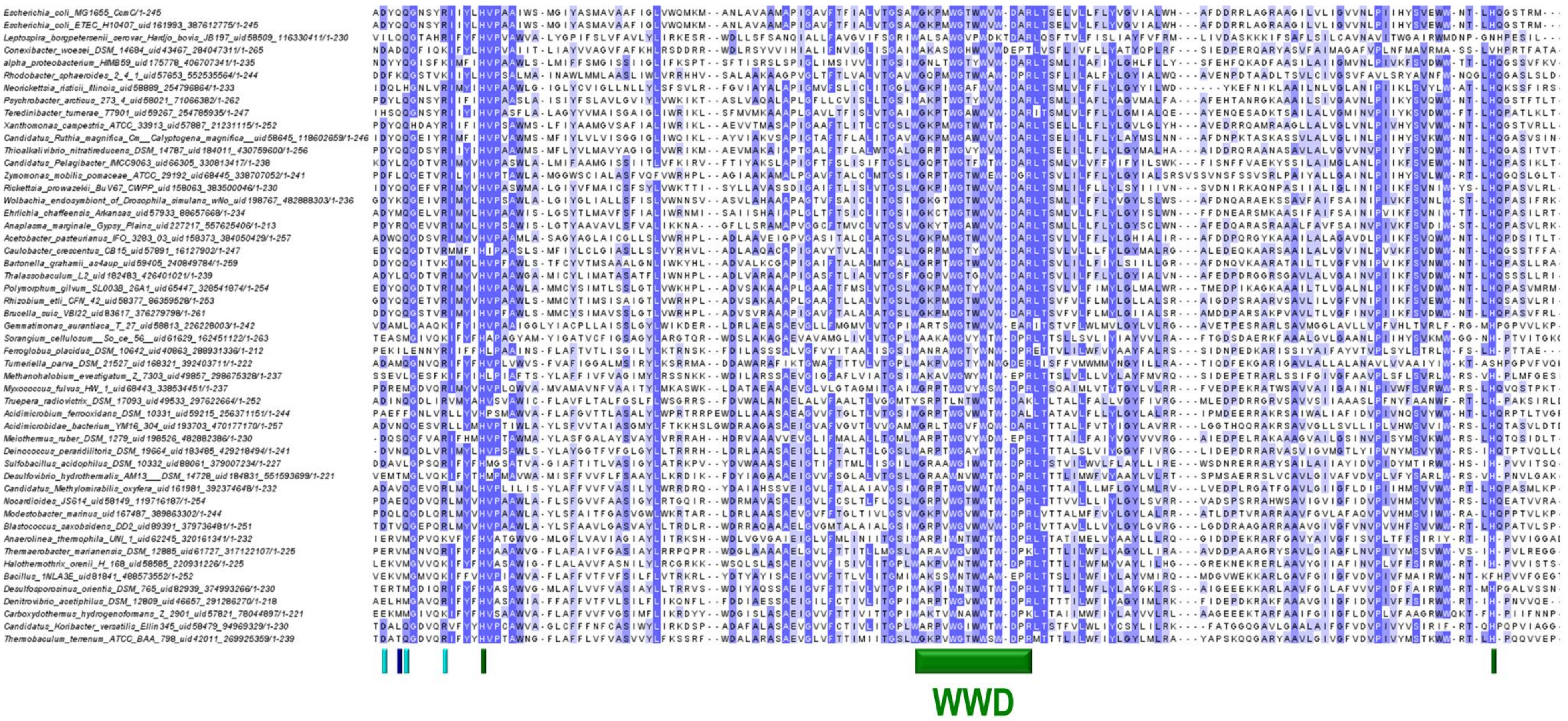

Figure S1. Residues involved in the interaction of CcmC with CcmE can be identified by bioinformatic analysis. The figure shows a sequence alignment of representative CcmC protein sequences from bacteria and archaea; only the most relevant part of the CcmC protein sequence is shown. The WWD motif of CcmC and the heme-ligating histidines (H60 and H184 in *E. coli*) are marked with green bars, residues potentially involved in the interaction with CcmE are marked with cyan bars and the position of Q49 in *E. coli* CcmC (which covaries with R104 of *E. coli* CcmE) is marked with a blue bar.

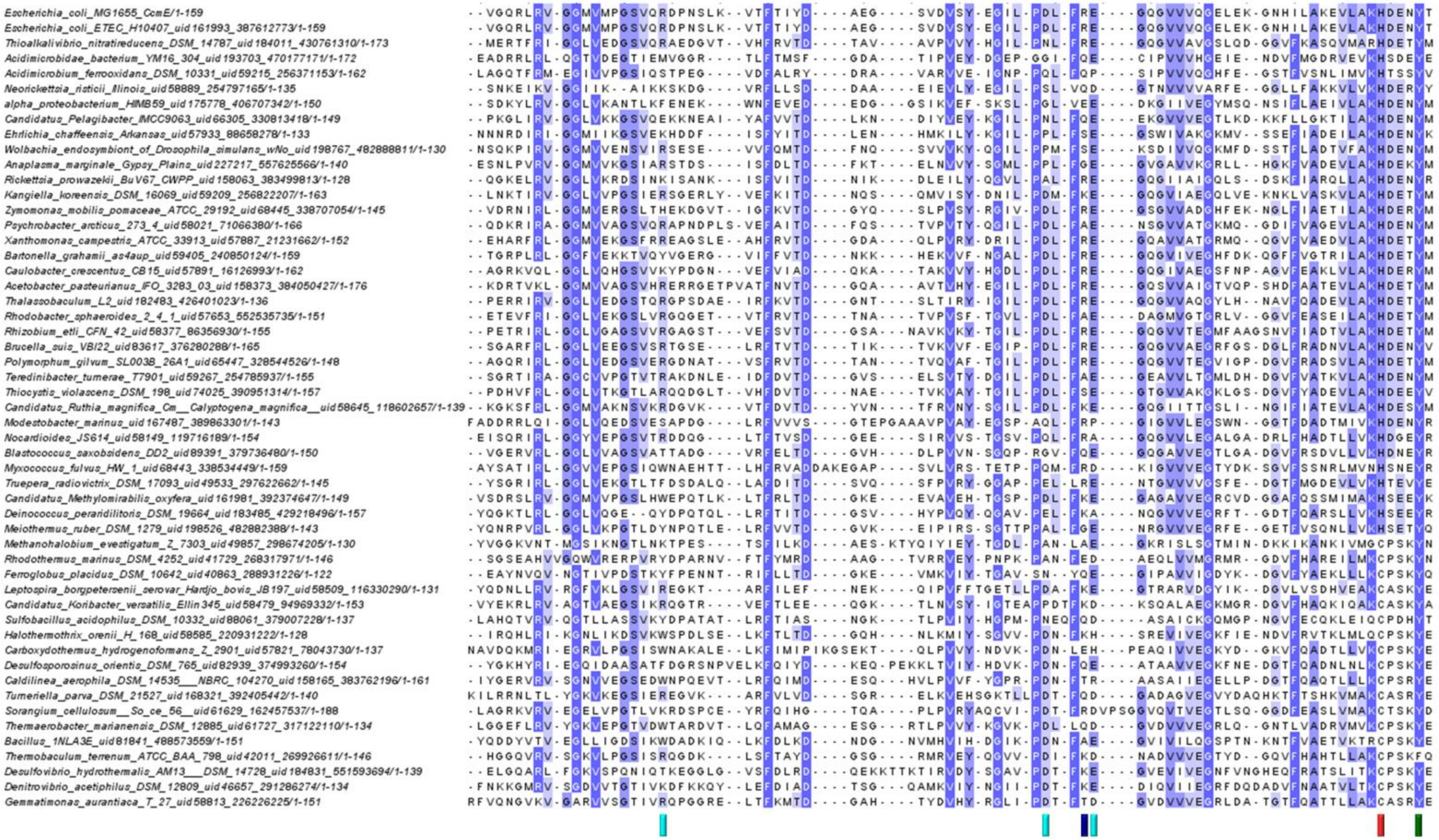

Figure S2. Residues involved in the interaction of CcmE with CcmC can be identified by bioinformatic analysis. The figure shows a sequence alignment of representative CcmE protein sequences from bacteria and archaea; only the most relevant part of the CcmE protein sequence is shown. The positions of the heme-binding residue (H130 in *E. coli* CcmE (1)) and its proposed axial ligand (Y134 in *E. coli* CcmE (2)) are marked with a red and a green bar, respectively, residues potentially involved in the interaction with CcmC are marked with cyan bars and the position of R104 in *E. coli* CcmE (which covaries with Q49 of *E. coli* CcmC) is marked with a blue bar.

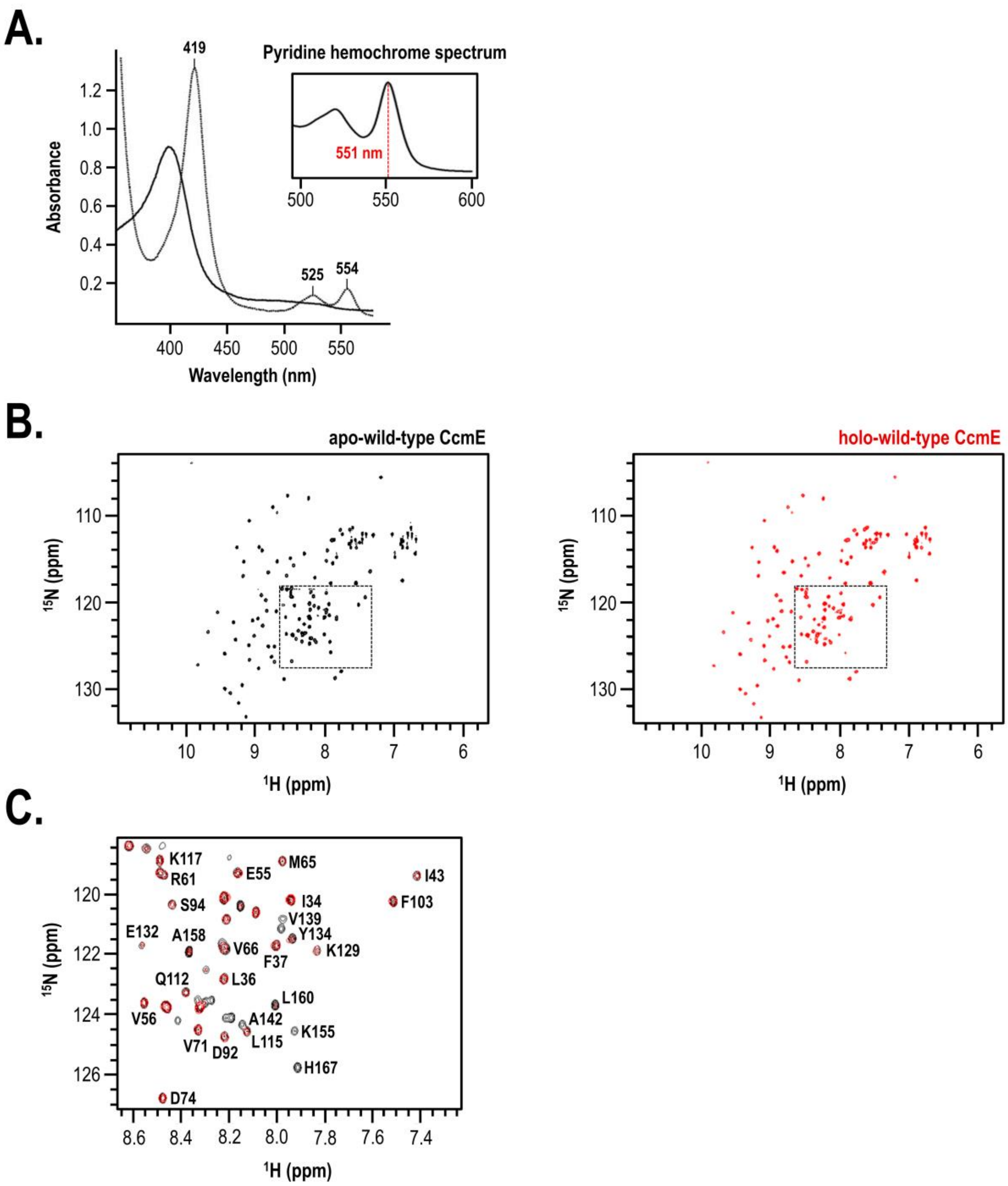

Figure S3. The integrity of leaderless wild-type *E. coli* CcmE, purified in the absence of System I proteins, was confirmed by, in addition to mass spectrometry, visible absorption spectroscopy and NMR spectroscopy. A) Visible absorption spectrum of oxidised (solid line) and reduced (dotted line) leaderless wild-type holo-CcmE showing identical spectral characteristics as *in vivo* produced holo-CcmE (1). The inset shows the reduced pyridine hemochrome spectrum of leaderless wild-type holo-CcmE with its characteristic α-band at 551 nm (1). B) ^{1}H-^{15}N HSQC spectra of ^{15}N-labelled leaderless wild-type apo-CcmE (left, black) and holo-CcmE (right, red) bearing a C-terminal His_6-tag collected at 750 MHz. This pair of spectra was used to generate the peak intensity ratios shown in Fig. 5A. The region outlined by a dashed box is expanded in panel (C). C) Overlay of a spectral region of apo- and holo-CcmE shown in panel (B), highlighting intensity changes observed because of the presence of covalently-bound paramagnetic heme. Most peaks are labelled with their resonance

assignments. The contour levels in the two spectra highlight the different behaviours observed for core residues (residues 33-130) versus residues at the C-terminus of the protein. Peaks for residues with an intensity ratio close to the average value in Fig. 5A show the same number of contours in the two spectra, while peaks for C-terminal residues with more pronounced broadening, show fewer or no contours in the holo-CcmE (red) spectrum. Changes in peak position are not observed between spectra of apo- and holo-CcmE; addition of heme only leads to peak broadening.

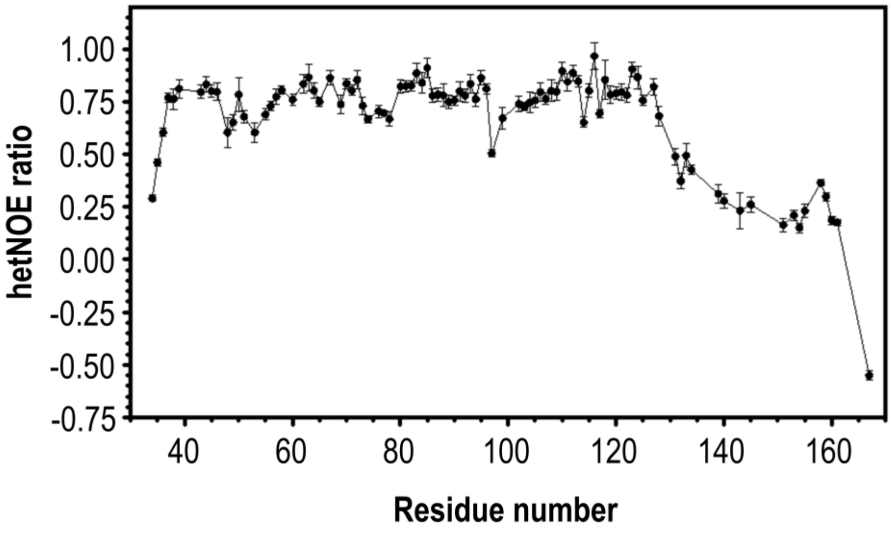

Figure S4. Apo-CcmE is flexible from residue 128 onwards. Experimental {^{1}H}-^{15}N heteronuclear NOE ratios for ^{15}N-labelled leaderless wild-type apo-CcmE bearing a C-terminal His$_6$-tag collected at 750 MHz. Errors derived from the Monte Carlo analysis of baseline noise are shown.

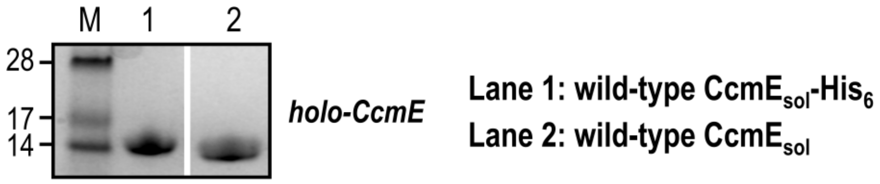

Figure S5. Covalent heme attachment during apo-CcmE reconstitution with heme occurs rapidly for protein concentrations required for NMR spectroscopy. 100 μM of leaderless wild-type apo-CcmE with (lane 1) and without (lane 2) a C-terminal His_6-tag were mixed with equimolar amount of heme at room temperature. The figure shows SDS-PAGE analysis of 15 μg of protein stained for covalently-bound heme ten minutes after heme addition. For both wild-type apo-CcmE constructs, intense heme-staining bands indicate significant covalent heme attachment by this timepoint of the reaction. Molecular weight markers (M) are on the left. Gaps indicate where a lane was removed.

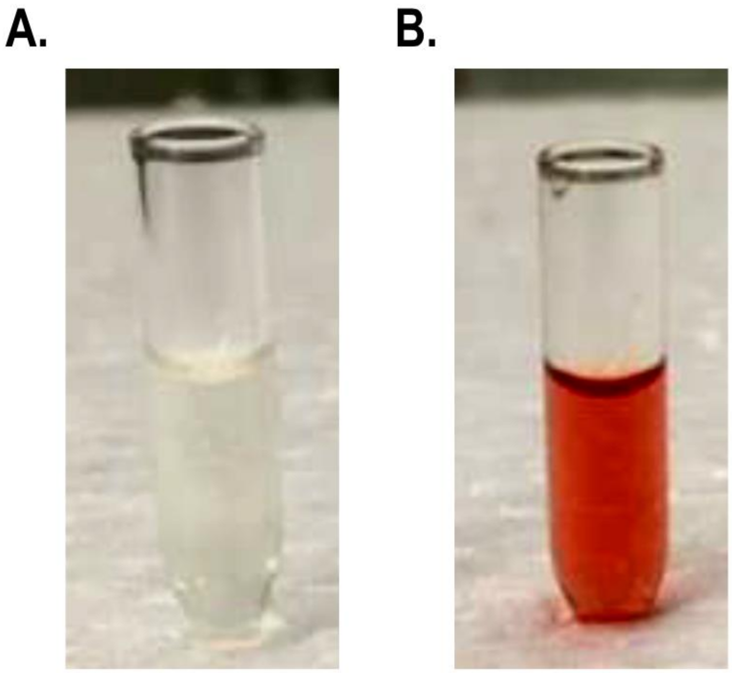

Figure S6. H130A-CcmE binds heme non-covalently and retains it during a rigorous wash process. A) H130A-apo-CcmE protein solution. B) H130A-CcmE protein solution after addition of excess heme and rigorous washing using a concentration device and multiple steps of concentration and re-dilution. The resulting protein solution contains heme and was used in NMR studies as a proxy of the of the pre-adduct state of CcmE, i.e. CcmE in the CcmC:heme:CcmE complex before covalent heme attachment.

SUPPORTING TABLES

Table S1. Bacterial strains used in this study.

**Name**	**Description**	**Source**
BL21 (DE3)<br>JCB387	F–, *omp*T, *hsd*S_B(r_B-, m_B-), *dcm*, *gal*, λ(DE3)<br>*E. coli* RV λ*nirB*	Stratagene<br>(3)

Table S2. Amino acids found in positions corresponding to Q49 and R104 of *E. coli* CcmC and CcmE, respectively, in other alpha and gamma-proteobacteria. These positions can be occupied by a broad range of residues, but ones that affect System I function (see Fig. 3), were not found to occur. The % frequency of occurrence of each pair (based on the number of organisms) is given in brackets.

**Residue at position Q49 of CcmC**	**Residue at position R104 of CcmE**	**Frequency of pair (no. of organisms)**
Q	R	582 [92.7%]
Q	K	9 [1.4%]
Q	S	3 [0.5%]
Q	A	1 [0.2%]
K	S	3 [0.5%]
K	A	2 [0.3%]
K	G	1 [0.2%]
K	D	1 [0.2%]
L	A	9 [1.4%]
L	R	4 [0.6%]
L	V	1 [0.2%]
R	K	1 [0.2%]
R	R	1 [0.2%]
V	K	4 [0.6%]
T	K	3 [0.5%]
Y	R	2 [0.3%]
G	K	1 [0.2%]

Table S3. Plasmids used in this study.

Name	Description	Source
pKPD1	*P. denitrificans* cytochrome c_{550}, Amp^R	(4)
pE221	Leaderless *E. coli* CcmE (S32-S163) with a C-terminal polyhistidine tag, pET28b(+) (Novagen), Amp^R	(5)
pSHS04	pE221 with a thrombin cleavage site before the C-terminal polyhistidine tag, Amp^R	This study
pSHS05	pSHS04 with a streptavidin II-tag after the C-terminal polyhistidine tag, Amp^R	This study
pSHS10	pSHS04 carrying the H130A mutation in *ccmE*, Amp^R	This study
pEC86	*E. coli ccmABCDEFGH*, pACYC184, Cam^R	(6)
pSHS32	pEC86 carrying the D47A mutation in *ccmC*, Cam^R	This study
pSHS24	pEC86 carrying the Q50A mutation in *ccmC*, Cam^R	This study
pSHS33	pEC86 carrying the R55A mutation in *ccmC*, Cam^R	This study
pSHS36	pEC86 carrying the R73A mutation in *ccmE*, Cam^R	This study
pSHS34	pEC86 carrying the D101A mutation in *ccmE*, Cam^R	This study
pSHS35	pEC86 carrying the E105A mutation in *ccmE*, Cam^R	This study
pSHS45	pEC86 carrying the R73A/D101A/E105A mutations in *ccmE*, Cam^R	This study
pSHS01	pEC86 carrying the Q49A mutation in *ccmC*, Cam^R	This study
pSHS02	pEC86 carrying the R104A mutation in *ccmE*, Cam^R	This study
pSHS03	pEC86 carrying the Q49A mutation in *ccmC* and the R104A mutation in *ccmE*, Cam^R	This study
pSHS11	pEC86 carrying the Q49R mutation in *ccmC* and the R104Q mutation in *ccmE*, Cam^R	This study
pSHS08	pEC86 carrying the Q49E mutation in *ccmC*, Cam^R	This study
pSHS12	pEC86 carrying the Q49I mutation in *ccmC* and the R104I mutation in *ccmE*, Cam^R	This study
pSHS17	pEC86 carrying the Q49S mutation in *ccmC*, Cam^R	This study
pSHS26	pEC86 carrying the R104S mutation in *ccmE*, Cam^R	This study
pSHS27	pEC86 carrying the Q49S mutation in *ccmC* and the R104S mutation in *ccmE*, Cam^R	This study
pSHS20	pEC86 carrying the Q49C mutation in *ccmC*, Cam^R	This study
pSHS21	pEC86 carrying the R104C mutation in *ccmE*, Cam^R	This study
pSHS22	pEC86 carrying the Q49C mutation in *ccmC* and the R104C mutation in *ccmE*, Cam^R	This study
pSHS28	pEC86 carrying the Q49K mutation in *ccmC* and the R104A mutation in *ccmE*, Cam^R	This study
pSHS16	pEC86 carrying the Q49A mutation in *ccmC* and the R104V mutation in *ccmE*, Cam^R	This study
pSHS37	pEC86 carrying the Q49V mutation in *ccmC* and the R104A mutation in *ccmE*, Cam^R	This study
pSHS38	pEC86 carrying the Q49V mutation in *ccmC* and the R104V mutation in *ccmE*, Cam^R	This study
pQE2-Im9	Modified TAGZyme pQE vector (pQE-2, Qiagen) encoding an N-terminal polyhistidine tag, followed by the gene of the immunity protein of colicin E9 from *E. coli* and a Factor Xa cleavage site before the multiple cloning site of TAGZyme pQE, Amp^R	This study
pCcmC1	*E. coli ccmC*, pQE2-Im9, Amp^R	This study
pSHS48	pCcmC1 carrying the D47AA mutation on *ccmC*, Amp^R	This study
pSHS49	pCcmC1 carrying the Q50A mutation on *ccmC*, Amp^R	This study
pSHS47	pCcmC1 carrying the R55A mutation on *ccmC*, Amp^R	This study

Table S4. Oligonucleotide primers for cloning used in this study.

Name	DNA sequence (5´-3´)
P1	GCGCATTAATATGGAACTGAAGCATAGCATTAGTGATTATACAG
P2	TCCCGCATGCATATGACGACCTTCGATCTGTTTAAATCCTGACTTACCG
P3	AAAAACATATGATGTGGAAAACACTGCATCAACTGG
P4	AAAAAAAGCTTTCATTTACGGCCTCTTTTCAGTATC

REFERENCES

1. Schulz, H., Hennecke, H., and Thony-Meyer, L. (1998) Prototype of a heme chaperone essential for cytochrome *c* maturation. *Science* **281**, 1197-1200

2. Uchida, T., Stevens, J. M., Daltrop, O., Harvat, E. M., Hong, L., Ferguson, S. J., and Kitagawa, T. (2004) The interaction of covalently bound heme with the cytochrome *c* maturation protein CcmE. *J. Biol. Chem.* **279**, 51981-51988

3. Hussain, H., Grove, J., Griffiths, L., Busby, S., and Cole, J. (1994) A seven-gene operon essential for formate-dependent nitrite reduction to ammonia by enteric bacteria. *Mol. Microbiol.* **12**, 153-163

4. Sambongi, Y., and Ferguson, S. J. (1994) Synthesis of holo *Paracoccus denitrificans* cytochrome c_{550} requires targeting to the periplasm whereas that of holo *Hydrogenobacter thermophilus* cytochrome c_{552} does not. Implications for *c*-type cytochrome biogenesis. *FEBS Lett.* **340**, 65-70

5. Daltrop, O., Stevens, J. M., Higham, C. W., and Ferguson, S. J. (2002) The CcmE protein of the *c*-type cytochrome biogenesis system: unusual *in vitro* heme incorporation into apo-CcmE and transfer from holo-CcmE to apocytochrome. *Proc. Natl. Acad. Sci. U. S. A.* **99**, 9703-9708

6. Arslan, E., Schulz, H., Zufferey, R., Kunzler, P., and Thony-Meyer, L. (1998) Overproduction of the *Bradyrhizobium japonicum* *c*-type cytochrome subunits of the cbb_3 oxidase in *Escherichia coli*. *Biochem. Biophys. Res. Commun.* **251**, 744-747
